# Health Assessment of Cooling Fan Bearings Using Wavelet-Based Filtering

**DOI:** 10.3390/s130100274

**Published:** 2012-12-24

**Authors:** Qiang Miao, Chao Tang, Wei Liang, Michael Pecht

**Affiliations:** 1 School of Mechanical, Electronic and Industrial Engineering, University of Electronic Science and Technology of China, Chengdu, Sichuan 611731, China; E-Mail: tchao_uestc@163.com (C.T.); zycqlw@126.com (W.L.); 2 Center for Advanced Life Cycle Engineering (CALCE), University of Maryland, College Park, MD 20742, USA; E-Mail: pecht@calce.umd.edu

**Keywords:** cooling fan, health assessment, prognostics and health management, comblet filtering, exponentially weighted moving average, health condition indicator

## Abstract

As commonly used forced convection air cooling devices in electronics, cooling fans are crucial for guaranteeing the reliability of electronic systems. In a cooling fan assembly, fan bearing failure is a major failure mode that causes excessive vibration, noise, reduction in rotation speed, locked rotor, failure to start, and other problems; therefore, it is necessary to conduct research on the health assessment of cooling fan bearings. This paper presents a vibration-based fan bearing health evaluation method using comblet filtering and exponentially weighted moving average. A new health condition indicator (HCI) for fan bearing degradation assessment is proposed. In order to collect the vibration data for validation of the proposed method, a cooling fan accelerated life test was conducted to simulate the lubricant starvation of fan bearings. A comparison between the proposed method and methods in previous studies (*i.e.*, root mean square, kurtosis, and fault growth parameter) was carried out to assess the performance of the HCI. The analysis results suggest that the HCI can identify incipient fan bearing failures and describe the bearing degradation process. Overall, the work presented in this paper provides a promising method for fan bearing health evaluation and prognosis.

## 1. Introduction

The integration level and energy consumption of electronic circuits is increasing, resulting in increased heat flux densities and temperatures in electronic devices. In addition, the recent trend toward super-light and super-thin electronics has imposed further challenges on system thermal design. According to [1], temperature has a great impact on electronic component reliability, and the failure rate of a component increases exponentially as the temperature increases. Therefore, it is necessary to utilize thermal design techniques so as to reduce the internal temperature of electronic devices. The working principle of thermal design is to accomplish the following: (1) lessen heat dissipation by utilizing low energy consumption techniques and reducing the number of heat-generating components; and (2) move heat out through conduction, convection, or radiation. Cooling fans, as an active heat transfer device, have been used in many electronics systems to lower the system temperature and improve reliability.

As a commonly used thermal solution for most electronic devices, cooling fans have simple structures with low cost. According to [2], cooling fan failure is a major problem for many electronic devices. It causes system instability, malfunctioning, and damage to electronic components by over-heating, and can finally lead to system failure [3]. This may result in severe economic, or even catastrophic, losses under certain applications, such as large-scale data servers in financial divisions, communication networks, avionics, medical devices, *etc.* Therefore, it is necessary to conduct research on cooling fan condition monitoring and health assessment to guarantee the normal operation of a fan.

A cooling fan is composed of both electronic and mechanical parts. The mechanical parts include the bearings, shaft, fan blades, and fan housing; out of these, bearing failure is the top contributor to fan failure. The types of bearings used in cooling fans can be categorized as sleeve bearings, ball bearings, fluid bearings, and magnetic bearings. The selection of bearings should consider parameters such as performance, durability, cost, size, weight, and noise. Ball bearings have the advantage of a good balance between these factors, and so they are widely used in cooling fans. Specifically, ball bearings have a longer lifespan at higher temperatures (63,000 hours at 50 °C) than sleeve bearings (40,000 hours at 50 °C) [3].

As a typical rolling-element bearing, a ball bearing is the fundamental rotating part in a mechanical system, and numerous studies have been conducted on bearing fault diagnosis [4–12]. Regarding the current progress on machinery health assessment, Miao *et al.* developed gear health assessment methods using empirical mode decomposition [13] and wavelet decomposition [14]. Wang *et al.* [15] presented gearbox fault diagnosis and prognosis by the fusion of multiple health indicators through support vector data description. Yang and Makis [16] used an ARX model to evaluate gearbox health conditions under variable load conditions. Lin *et al.* [17] proposed an approach for gearbox condition-based maintenance, and the fault growth parameter was defined using the residual error signal. Qiu *et al.* [18] proposed a self-organizing-map-based performance degradation method for assessing bearing health condition. Ocak *et al.* [19] developed a new scheme based on wavelet packet decomposition and the hidden Markov model for bearing prognostics. Pan *et al.* [20,21] used wavelet packet node energies as bearing fault features. Then, fuzzy c-means [20] and support vector data description [21] were respectively employed to evaluate how far the current bearing health condition was from normal bearing health condition. In their following up studies, Pan *et al.* [22] proposed a hybrid model for bearing performance degradation utilizing support vector data description and fuzzy c-means. Jiang *et al.* [23] proposed a new approach combining the autoregressive model and fuzzy cluster analysis for bearing diagnosis and degradation assessment. Shen *et al.* [24] considered the cumulative characteristics of bearing performance deterioration and proposed a monotonic health index for evaluating bearing health condition. Lei *et al.* [25] proposed health indicators for monitoring planetary gearboxes health condition.

The purpose of this research is to investigate cooling fan bearing health assessment methods and develop a prognostics and health management (PHM) solution for fan degradation assessment. However, the literature on fan bearing health assessment is limited. The Center for Advanced Life Cycle Engineering (CALCE) at the University of Maryland, has conducted research on fan bearings, including fan bearing fault identification [3], a physics-of-failure approach for fan PHM [26], a precursor monitoring approach for cooling fans [27], and fan bearing degradation using acoustic emission [28]. This paper proposes a new health indicator for fan bearing degradation assessment. The comblet, which was initially proposed by Miller [29] for gearbox vibration analysis, is utilized for the extraction of fault-sensitive information from the frequency domain of the bearing vibration signal. The health indicator is defined by data taken from the bearing vibration spectrum, incorporating the idea of exponentially weighted moving average (EWMA). The proposed EWMA based health indicator can utilize historical information (current and previous data) about the test sample, and it does not require model training, as opposed to other related studies [18–22,24]. To validate the proposed method, a test rig for a cooling fan accelerated life test was established, and a set of fan bearing vibration data collected from the test rig was used.

The rest of this paper is organized as follows: Section 2 introduces the fundamentals of the comblet filter. In Section 3, a new health indicator for fan bearing degradation assessment is proposed. In Section 4, the fan bearing accelerated life test rig is introduced, and then vibration data collected from this test rig are used for validation of the proposed method. Conclusions are presented in Section 5.

## 2. Principle of Comblet Filtering

### 2.1. Time-Domain Synchronous Averaging

Time-domain synchronous averaging (TSA) is a useful technique for rotating machinery fault diagnosis. It can extract fault-related periodic information from complicated signals and eliminate extraneous periodic components and noise. In TSA, the measured signal is synchronously averaged over the rotational period of the target of interest. The nonsynchronous vibration from other sources and noise are averaged out by applying this procedure. After a large amount of averaging in the time-domain, the averaged signal gradually approximates the expected periodic signal, and the signal to noise ratio is improved.

Given a piece of signal *s*(*t*), the corresponding time-domain averaging *s̄*(*t*) can be defined as:

(1) $$\bar{s}(t)=\left(\frac{1}{N}\right)\sum_{m=0}^{N-1}s(t-mT)$$

where *T* is the period of the target component, and *N* is the number of averages.

Taking the Z-transform on Equation (1), the following can be obtained:

(2) $$\bar{S}(z)=\left(\frac{S(z)}{N}\right)\sum_{m=0}^{N-1}z^{-mT}$$

where *S*(*z*) is the Z-transform of *s*(*t*).

Thus, the transfer function of the time-domain averaging is obtained as:

(3) $$H(z)=\frac{\bar{S}(z)}{S(z)}=\left(\frac{1}{N}\right)\left(\frac{1-z^{-NT}}{1-z^{-T}}\right)$$

The frequency response of Equation (3) can be calculated as:

(4) $$H({\text{e}}^{\text{j}\omega })=\left(\frac{1}{N}\right)\left(\frac{\sin\left(\frac{NT\omega }{2}\right)}{\sin\left(\frac{T\omega }{2}\right)}\right)\left({\text{e}}^{\text{j}\omega (N-1)\frac{T}{2}}\right)$$

The corresponding amplitude response and phase responses are described as:

(5) $$\left|H({\text{e}}^{\text{j}\omega })\right|=\left(\frac{1}{N}\right)\left(\frac{\sin\left(\frac{NT\omega }{2}\right)}{\sin\left(\frac{T\omega }{2}\right)}\right)$$

(6) $$\arg(H({\text{e}}^{\text{j}\omega }))=-\omega (N-1)\frac{T}{2}$$

Therefore, TSA can be seen as a comb impulse train filter which implements signal filtering in the frequency domain. It keeps the information around the main frequency and its harmonics and suppresses other unrelated frequency components. However, good performance of the TSA technique requires many averages and long signal lengths, and these may not be available due to cost and other technical restrictions in data collection. Furthermore, the underlying assumption of the constant rotation speed is not always met in rotating systems because the rotation speed fluctuates according to working conditions, such as load and electrical supply. If the rotation speed is fluctuating, synchronous sampling is necessary, which collects vibration at a rate related directly to the rotation speed of the target. Another solution is to record the vibration signal at an arbitrary sampling rate and do resampling through interpolation. However, the implementation of these techniques is difficult due to the higher cost of hardware and increased computational burden.

### 2.2. Wavelet Filtering

The wavelet transform provides a time-frequency representation of a signal through a set of wavelet basis functions [30]. It has been widely used in machinery fault diagnosis. Given a mother wavelet function, *ψ*(*t*), a series of wavelet functions {*ψ_a,b_*(*t*)} can be defined as:

(7) $$\psi_{a,b}(t)={\left|a\right|}^{\overset{-1}{\;}/\underset{2}{\;}}\psi (\frac{t-b}{a}),a,b\in R,a\neq 0$$

where *a* is the scale parameter, *b* is the translation parameter, and *R* represents a set of real numbers.

The wavelet function *ψ*(*t*) should satisfy the following admissibility criterion:

(8) $$C_{\psi }=\int_{R}\frac{{\left|\psi (\omega )\right|}^{2}}{\left|\omega \right|}\text{d}\omega <\infty$$

where *ψ*(*ω*) denotes the Fourier transform of *ψ*(*t*). The continuous wavelet transform of a signal *x*(*t*) can be described as:

(9) $$W(a,b)=\langle x(t),\psi_{a,b}(t)\rangle =|a{|}^{-1/2}\int_{R}x(t)\psi^{*}(\frac{t-b}{a})\text{d}t$$

where ψ*(*t*) represents the complex conjugation of *ψ*(*t*).

The equivalent frequency-domain representation can be expressed as:

(10) $$WT(a,b)=\sqrt{a}F^{-1}\left[X(f)\Psi^{*}(af)\right]$$

where *X*(*f*) and Ψ(*f*) are the Fourier transforms of *x*(*t*) and *ψ*(*t*), respectively, and *F*^−1^ represents the inverse Fourier transform.

Accordingly, the continuous wavelet transform can be treated as a band-pass filter. The bandwidth and central frequency of the filter is determined by the scale parameter *a* of the wavelet function.

### 2.3. Comblet Filter Design

The wavelet coefficient *W*(*a*,*b*) measures the correlation between the wavelet function and the signal of interest at different frequencies determined by the scaling parameter *a* and at different time locations determined by the translation parameter *b*. A coefficient with a large value means that the correlation between the wavelet function and the signal is high; conversely, a small value indicates a low correlation. Thus, the wavelet function can be designed according to the characteristics of the signal.

Due to its property of time-frequency localization, the Morlet wavelet has been widely used in signal processing. It is defined as:

(11) $$\psi (t)=\beta {\text{e}}^{-\sigma^{2}t^{2}+\text{j}2\pi f_{0}t}$$

where *σ* is the shape factor, *f*_0_ is the wavelet central frequency, and *β* is a positive parameter.

The Fourier transform of the Morlet wavelet is:

(12) $$\Psi (f)=\frac{\beta \sqrt{\pi }}{\sigma }{\text{e}}^{-{\left(\frac{\pi }{\sigma }\right)}^{2}{\left(f-f_{0}\right)}^{2}}$$

**Figure 1.**
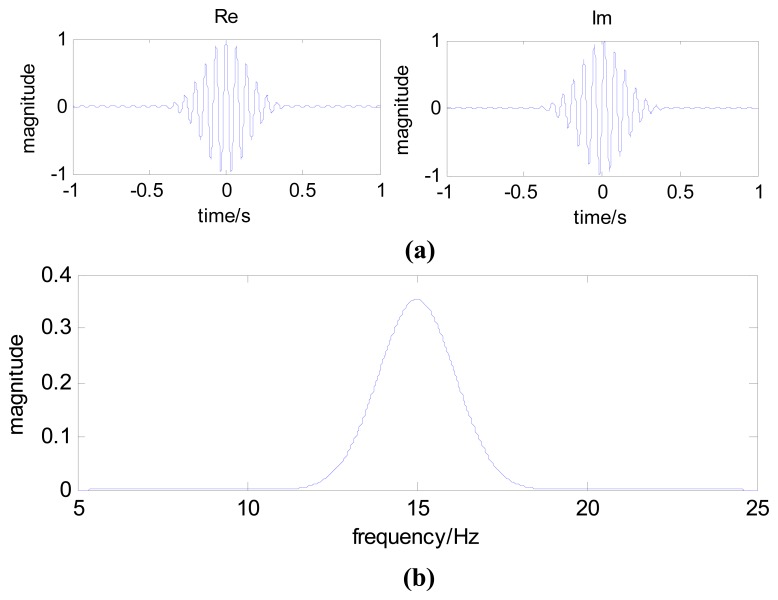
(**a**) Time-domain plot of the complex Morlet wavelet; (**b**) Frequency-domain plot of the complex Morlet wavelet.

Figure 1 shows the time-domain and frequency-domain plots of a complex Morlet wavelet, given that *f*_0_=15 Hz, *σ*=5, and *β*=1.

As seen in Section 2.1, TSA can be treated as a comb filter that extracts fault-related information from a complicated vibration signal. To overcome the aforementioned limitations of TSA, a new wavelet function is designed which possesses the properties of both the exponential decay of the Morlet wavelet and the flat passband of the harmonic wavelet. The new wavelet is called a comblet, and the mathematic definition of this wavelet is given by:

(13) $$\Psi_{1}(f)=\{\begin{array}{cc} \frac{\beta \sqrt{\pi }}{\sigma }{\text{e}}^{-{\left(\frac{\pi }{\sigma }\right)}^{2}{\left[f-(f_{0}-\text{bw})\right]}^{2}} & f\leq f_{0}-\text{bw}\\ \frac{\beta \sqrt{\pi }}{\sigma }{\text{e}}^{-{\left(\frac{\pi }{\sigma }\right)}^{2}{\left[f-(f_{0}+\text{bw})\right]}^{2}} & f\geq f_{0}+\text{bw}\\ 1 & f_{0}-\text{bw}<f<f_{0}+\text{bw} \end{array}$$

where *f*_0_ is the comblet central frequency and bw is the half central bandwidth. Typically, *β* is chosen as $\frac{\sigma }{\sqrt{\pi }}$, and Equation (13) can be re-written as:

(14) $$\Psi_{1}(f)=\{\begin{array}{cc} {\text{e}}^{-{\left(\frac{\pi }{\sigma }\right)}^{2}{\left[f-(f_{0}-\text{bw})\right]}^{2}} & f\leq f_{0}-\text{bw}\\ {\text{e}}^{-{\left(\frac{\pi }{\sigma }\right)}^{2}{\left[f-(f_{0}+\text{bw})\right]}^{2}} & f\geq f_{0}+\text{bw}\\ 1 & f_{0}-\text{bw}<f<f_{0}+\text{bw} \end{array}$$

The half central bandwidth is defined as:

(15) $$\text{bw}=\frac{1}{2}c\cdot f_{0}$$

Here, *c* is the rotation variation parameter, which describes the percentage of rotation fluctuation.

**Figure 2.**
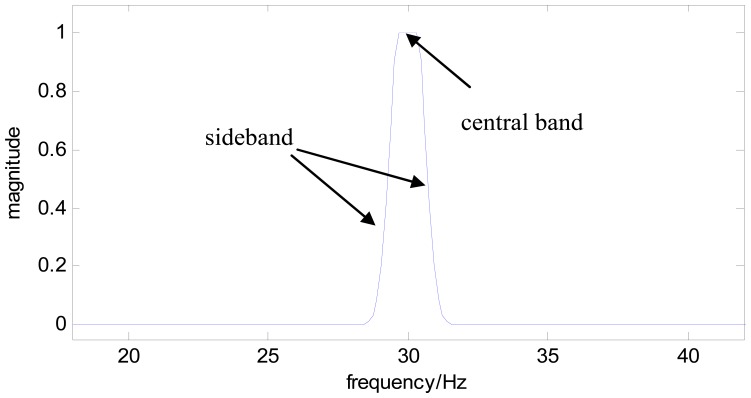
Frequency-domain plot of a comblet with one tooth.

In bearing fault diagnosis, the comblet central frequency, *f*_0_, is usually selected as the fault-related characteristic frequency *f_c_*. Figure 2 gives an example of this comblet function in the frequency domain with one comb tooth, where the central band has a magnitude of 1 (*i.e.*, $\beta =\frac{\sigma }{\sqrt{\pi }}$), the rotation variation parameter *c* is 2%, and the sideband follows the Morlet wavelet function.

From Equation (14), the comblet filter can be designed by constructing a comb filter where the comb teeth correspond to the fault-related characteristic frequency and its harmonics. According to the Nyquist-Shanon sampling theorem, the bandwidth limit *B* of a signal is determined by the sampling frequency *f_s_*. That is:

(16) $$B\leq f_{s}/2$$

Thus, the maximum number of comb teeth in a comblet filter is obtained by:

(17) $$n=\left\lfloor 0.5f_{s}/f_{c}\right\rfloor$$

where *f_c_* is the fault-related characteristic frequency and ⌊ ● ⌋ denotes the round-down operation. Therefore, a comblet filter with *n* comb teeth is written as:

(18) $$C_{f}(f)=\sum_{i=1}^{n}\Psi_{1}\left[f-\left(i-1\right)f_{c}\right]$$

**Figure 3.**
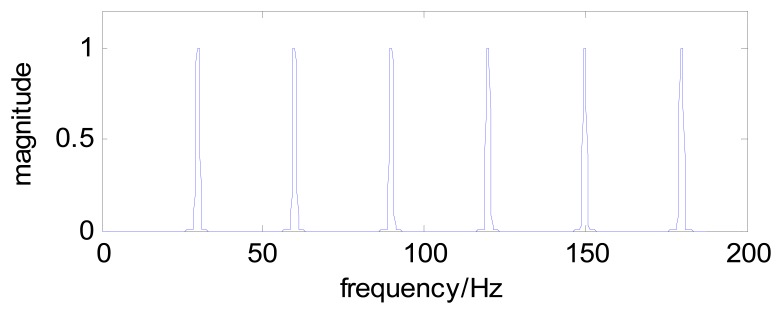
The frequency-domain plot of a comblet filter with *n*=6 comb teeth.

**Figure 4.**
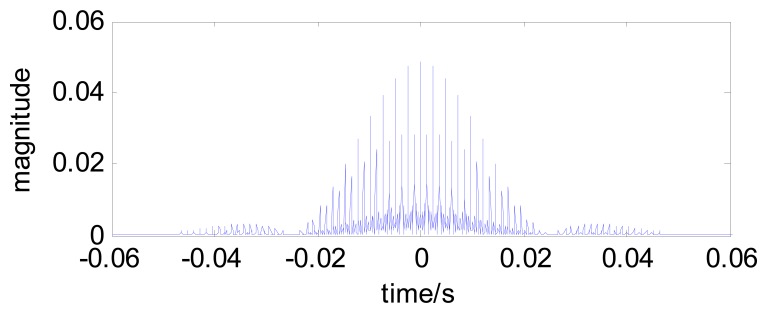
The time-domain plot of a comblet filter with *n*=6 comb teeth.

For example, a comblet filter with *n*=6 comb teeth can be constructed based on the single tooth comblet shown in Figure 2. The new comblet filter is shown in Figure 3, and Figure 4 is the time-domain plot of this comblet filter.

## 3. Proposed Fan Bearing Health Assessment Method

### 3.1. Fan Bearing Vibration Characteristics

A vibration signal collected from a cooling fan involves the integration of several components, including shaft, bearing, motor, blade, and noise. If there is a local defect on a certain part of a bearing, an impulse is generated when a mating element encounters the local defect. Since the local defect iteratively contacts with other parts of the bearing, it generates low-frequency vibration components. The frequency of the vibration signal is related to the rotation speed and the geometrics of the bearing; it is called the bearing characteristic frequency (BCF). Typical failures of ball bearings include local defects on the rolling element, inner race, outer race, and cage. The corresponding bearing characteristic frequencies are defined as follows [3]:

Ball spin frequency (BSF):

(19) $$\text{BSF}=f_{\text{cR}}=\frac{Df_{r}}{2d}[1-{\left(\frac{d}{D}\cos\gamma \right)}^{2}]$$

Ball pass frequency, inner race (BPFI):

(20) $$\text{BPFI}=f_{\text{cI}}=\frac{mf_{r}}{2}(1+(\frac{d}{D}\cos\gamma )$$

Ball pass frequency, outer race (BPFO):

(21) $$\text{BPFO}=f_{\text{cO}}=\frac{mf_{r}}{2}(1-(\frac{d}{D}\cos\gamma )$$

Fundamental train frequency (FTF):

(22) $$\text{FTF}=f_{\text{cC}}=\frac{f_{r}}{2}(1-(\frac{d}{D}\cos\gamma )$$

where *f_r_* is the bearing rotation speed (Hz), *m* is the number of rolling elements, *d* is the mean diameter of the rolling elements (mm), *D* is the pitch diameter of the bearing (mm), and *γ* is the contact angle (°).

It should be noted that the bearing characteristic frequencies are non-integer multiples of the rotation speed. In practice, since the rolling motions are accompanied by a degree of sliding which occurs in the contact areas [7], the derived bearing characteristic frequencies (Equations (19)–(22)) are approximate. The resulting variation in bearing frequency is typically around 1–2% [7], which provides a criterion for the choice of rotation variation parameter *c*.

### 3.2. Implementation of Comblet Filtering

As previously mentioned, vibrations produced by a cooling fan can be complex and can result from many different sources including the shaft, bearing, motor, blade, and noise. These different vibration sources interact with each other, which makes it almost impossible to identify the frequency of interest from the vibration spectrum without any data manipulation methods. In particular, vibration generated by a local defect in a bearing is usually very weak at the initial failure stage, and fault-related information, such as BCFs, is masked by other vibrations. It is therefore advantageous to remove the irrelevant information before proceeding with bearing health assessment.

A wavelet transform can be seen as a kind of bandpass filtering operation on a signal. The central frequency and bandwidth of the wavelet are tuned by the scale parameter *a*. In the design of the comblet filter, the central frequency and bandwidth are determined by *f*_0_ and *c*, and these parameters can be changed according to the practical scenario. Therefore, the comblet filtering technique provides a flexible solution for the extraction of bearing-fault-related information.

As stated in Section 2.3, the comblet is a new wavelet defined on the basis of some classical wavelet functions. Thus, the comblet transform can be described as the filtering operation on the signal *x*(*t*) under consideration with a comblet filter. Mathematically, it is defined as:

(23) $$W_{c}(\tau )=\langle x(t),c_{f}(t)\rangle =\int_{R}x(t){c_{f}}^{*}(t-\tau )\text{d}t=F^{-1}\left[X(f)C_{f}(f)\right](\tau )$$

where *W_c_*(*τ*) is the comblet coefficient in which *τ* is the translation parameter, 〈 ● 〉 represents the convolution operation, * is the complex conjugation operation, *C_f_*(*f*) is the constructed comblet function, and *c_f_*(*t*) is the inverse Fourier transform of the comblet. After comblet filtering, a time-domain filtered signal *W_c_*(*τ*) is obtained, and further analysis can proceed.

### 3.3. Fan Bearing Degradation Assessment

Before discussing methods for fan bearing degradation assessment, it is important to have an understanding of how the vibration signal changes as bearing failures develop. In general, bearing failure progresses through pre-failure, early failure, near failure, and near catastrophic failure stages [31]. In this process, the size of a local defect in a bearing becomes larger, and the impulses excited by the local defect are intensified. Therefore, the energy of the vibration signal tends to enhance around the BCFs and their harmonics, and the health indicator can be constructed to describe the fan bearing health condition.

Given the filtered signal *W_c_*(*τ*), the spectrum analysis is performed by:

(24) $$S_{c}(f)=\left|\int_{-\infty }^{+\infty }\left|W_{c}(\tau )\right|{\text{e}}^{-i2\pi f\tau }\text{d}\tau \right|$$

where *S_c_*(*f*) denotes the absolute value of the Fourier transform amplitude of the filtered signal *W_c_*(*τ*).

Assume the spectrum energy of the filtered signal is *E*. The definition of *E* is given by:

(25) $$E=20\log_{10}\left(\sum_{1}^{L}S_{c}(f)\right)$$

where *L* is the length of *S_c_*(*f*).

In order to detect the occurrence of fan bearing incipient failures, an exponentially weighted moving average (EWMA) is utilized to define the health indicator. The exponentially weighted moving average is very effective in detecting small shifts in the process [32], and it is suitable for bearing incipient failure detection since a failure symptom is very weak initially.

We chose the spectrum energy *E_t_* as the observation at time *t* in the process. The first *k* observations are used to estimate the initial health condition indicator:

(26) $${\text{HCI}}_{0}=\frac{1}{k}\sum_{i=1}^{k}E_{i}$$

After that, the remaining observations are used to evaluate the fan bearing health condition. The length of the observation sequence for evaluation is *l*. The proposed health condition indicator (HCI) is calculated as:

(27) $${\text{HCI}}_{t}=\lambda E_{t}+(1-\lambda ){\text{HCI}}_{t-1},t=1,2,3,\ldots ,l$$

where 0 < *λ* ≤ 1 is the weight given to the historical data. A large *λ* gives more weight to recent data and less weight to older data. The value of *λ* is usually set between 0.2 and 0.3 [32], and in this paper it is selected as *λ* =0.3.

**Figure 5.**
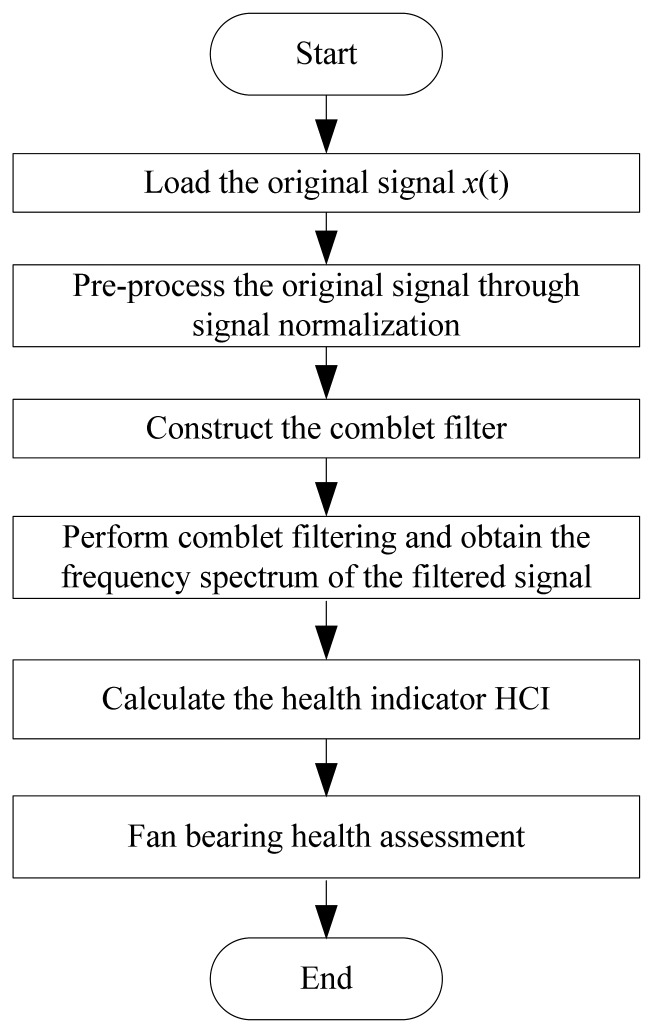
Flow chart of the proposed cooling fan bearing health assessment method.

Figure 5 presents a flow chart of the cooling fan bearing health assessment method presented in this paper. Given the original signal collected from the cooling fan, pre-processing is conducted to normalize the data. The comblet filters are designed using the bearing characteristic frequencies, including BSF, BPFI, BPFO, and FTF. After performing comblet filtering, the frequency spectrum is obtained. The health indicator HCI can be calculated to assess the fan bearing health condition.

## 4. Case Study with Fan Bearing

### 4.1. Description of Experimental Setup

Generally, the lifespan of a cooling fan can be several years, and it is uneconomical to conduct a life test under nominal working conditions. For a cooling fan working under its nominal load, lubrication degradation leads to wear in the bearing and shortens the lifespan of the cooling fan. Therefore, it is reasonable to choose the lubrication level to simulate lubrication degradation and accelerate the fan bearing life test. Fan bearing lubrication usually includes grease and oil from the manufacturing process. Assuming that the nominal amount of grease is at the 100% level, a certain lubrication level p% represents the percentage of grease being added to the bearing.

**Figure 6.**
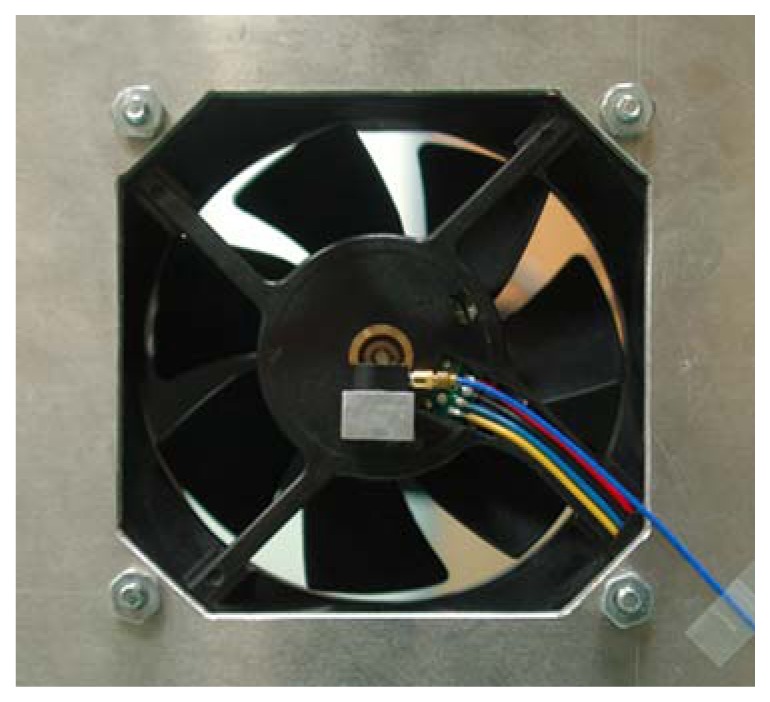
The BLDC cooling fan tested in this experiment.

The cooling fan used in this research was an axial type brushless direct current (BLDC) fan with dimensions of 92 × 92 × 38 mm. The fan has two ball bearings to support the shaft, whose overall diameter is 8 mm. Figure 6 shows the cooling fan tested in this experiment. The geometric specifications of the fan bearing used in this experiment are given in Table 1.

In the experiment, a cooling fan with bearings containing only residual oil and no added grease (0% lubrication level) was used in the life test. To measure the vibration data, an in-situ monitoring system was established with a PCB 352C42 accelerometer attached to the fan housing near the bearing. Since a higher temperature reduces the film thickness between mating surfaces, thus accelerating localized deformation and friction on the bearing mating components, the fan was stressed in a chamber at a temperature of 70 °C. The rotation speed of the fan was 4800 rpm, corresponding to a frequency, *f_r_*, of 80 Hz. A condenser microphone was set up 50 cm away from the cooling fan to record the acoustic noise from the fan. Data collection was conducted using the National Instruments LabVIEW program. The test was stopped when the acoustic noise increased 3dB from the initial value, which is one of fan failure criteria defined in the IPC-9591 standard [33]. Given the fan rotation speed, the bearing characteristic frequencies were calculated, as listed in Table 2.

The cooling fan was in a good health condition before the experiment. The accelerated life test started at 09/09/2010 10:14 and ended at 09/22/2010 0:47, when the acoustic noise measured by the microphone increased 3dB from its initial value. In this period, the experiment paused occasionally. The vibration signal was collected in blocks of 10 seconds every 15 minutes, and the sampling frequency, *f_s_*, was 25.6 kHz. The vibration signal was saved as a data file and numbered sequentially. There were a total of 388 data files. Since the first data file was a measurement during oven stabilization, it was excluded from the data analysis. Thus, the data set used in this paper included 387 data files.

### 4.2. Evaluation of the Proposed Health Indicator

To validate the proposed fan bearing health assessment method, the comblet filters were designed first. The central frequency *f*_0_ of each BCF filter is the corresponding bearing characteristic frequency. The rotation variation parameter *c* is selected as 2%. The half central bandwidth bw and the number of comb teeth in each comblet filter are calculated using Equations (15) to (17). The calculation results are listed in Table 3.

**Figure 7.**
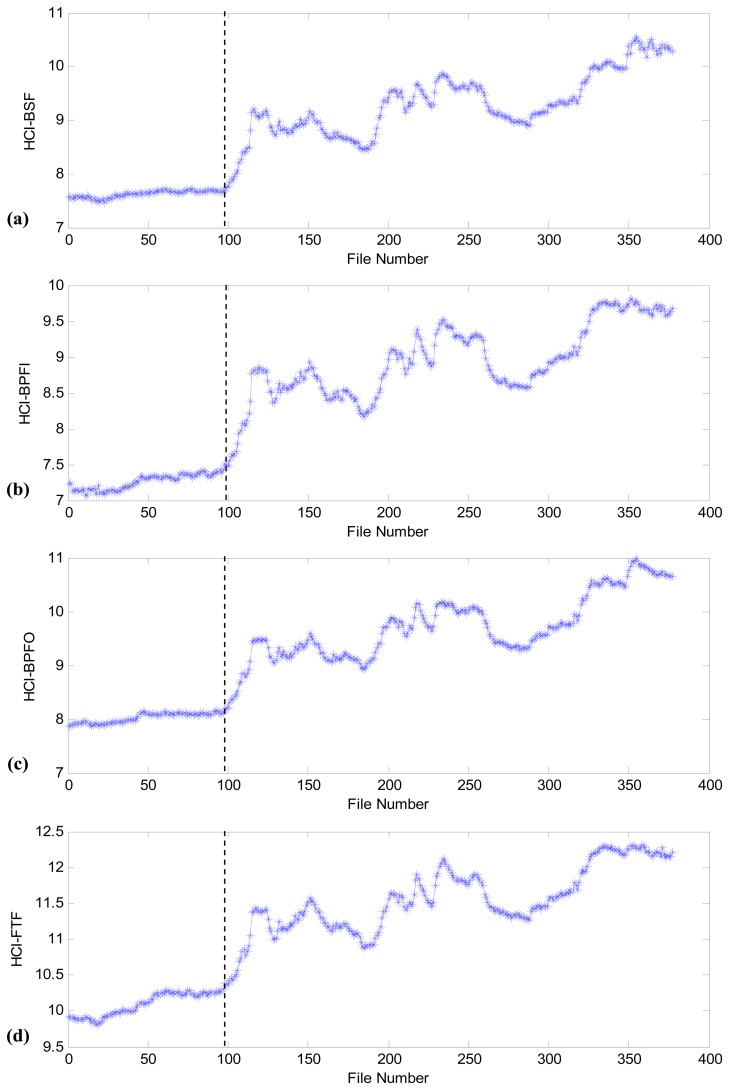
Fan bearing health assessment using the proposed HCI with different BCFs: (**a**) BSF; (**b**) BPFI; (**c**) BPFO; (**d**) FTF.

After the design of the comblet filters, the proposed health indicator is validated using the collected data set. The first 10 data files are used to calculate the initial health condition indicator, HCI_0_. The remaining 377 data files are used for the fan bearing health assessment and are numbered as 1 to 377. Figure 7 shows the assessment results with the four comblet filters. From Figures 7(a–d), it can be observed that the incipient failure should occur around file number 98–99, which corresponds to the time at 09/10/2010 20:36. The dashed line in Figure 7 represents the start of the fan bearing incipient failure.

**Figure 8.**
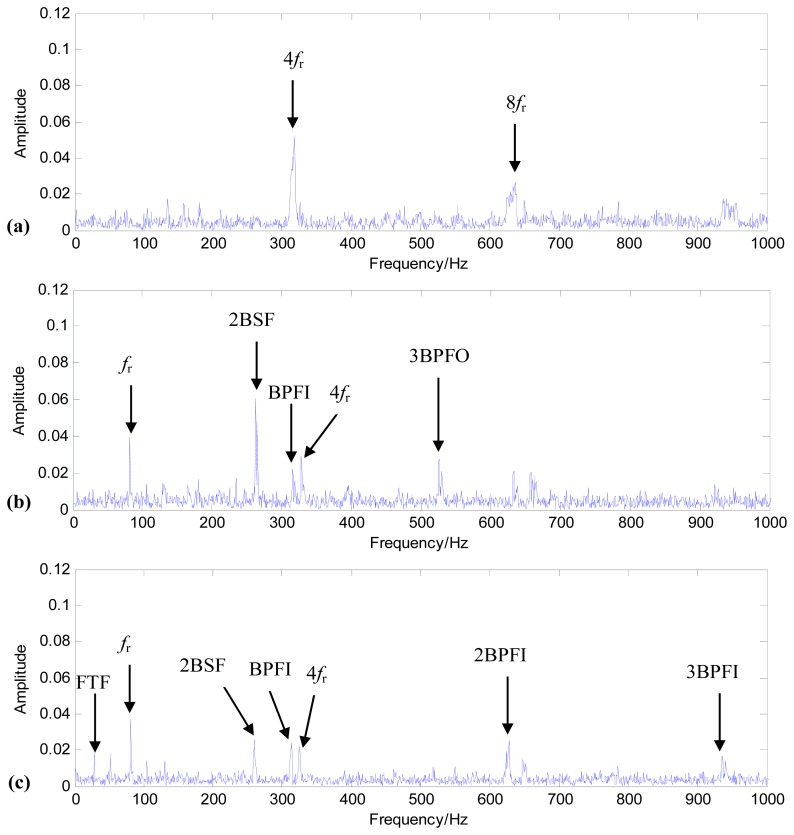
The spectral analysis of vibration data files: (**a**) the spectrum of data file 97; (**b**) the spectrum of data file 98; (**c**) the spectrum of data file 99.

In order to verify that the fan bearing incipient failure started at file numbers 98–99, Fourier spectrum analysis was conducted on data files 97, 98, and 99 after filtering. Figure 8 shows the spectral analysis results. Figure 8(a) is the spectrum of data file 97, and only the 4th and 8th order harmonics of the rotation frequency, *f_r_*, can be identified. From Figures 8(b,c), the four BCFs (BSF, BPFI, BPFO, FTF) and their harmonics can be identified from the spectral analysis results. Based on the results in Figure 8, it can be concluded that the bearing incipient failures started at data files 98–99.

### 4.3. Comparison with Other Health Indicators

In this section, a comparison study is conducted between the proposed HCI and other methods. In vibration analysis, the root mean square (RMS) and kurtosis are two popular statistics of the time-domain signal *γ*(*t*) for fault diagnosis, and they are given by:

(28) $$x_{\text{RMS}}=\sqrt{\frac{1}{T}[y^{2}(1)+y^{2}(2)+\ldots +y^{2}(T)]}$$

(29) $$\text{Kurtosis}=\frac{\sum_{t=1}^{T}{\left(y(t)-\bar{y}\right)}^{4}}{(T-1)\sigma^{4}}$$

where *y*(*t*), *t* = 1, 2,…, *T* is the sampling point of the signal, *T* is the number of sampling points, *ȳ* is the mean of the signal *y*(*t*), and *σ* is the standard deviation of the signal.

Another health indicator is fault growth parameter 1 (FGP1) [14]. It is defined as the part (percentage of points) of the residual error signal that exceeds three standard deviations calculated from the baseline residual error signal:

(30) $$\text{FGPI}=100\sum_{i=1}^{L}\frac{w_{i}}{W}I(r_{i}>\bar{r}+3\sigma_{0})$$

(31) $$w_{i}=I(r_{i}\leq \bar{r}+3\sigma_{0})+\left(\left\lfloor \frac{r_{i}-\bar{r}}{3\sigma_{0}}\right\rfloor -1\right)I(r_{i}>\bar{r}+3\sigma_{0}),W=\sum_{i=1}^{L}w_{i}$$

where the *r_i_*'s are the current residual error signal points, *r̄* is the mean of the current residual signal, *σ*_0_ is the “baseline” standard deviation, *I*● is the indicator function, and ⌊●⌋ is the floor function.

**Figure 9.**
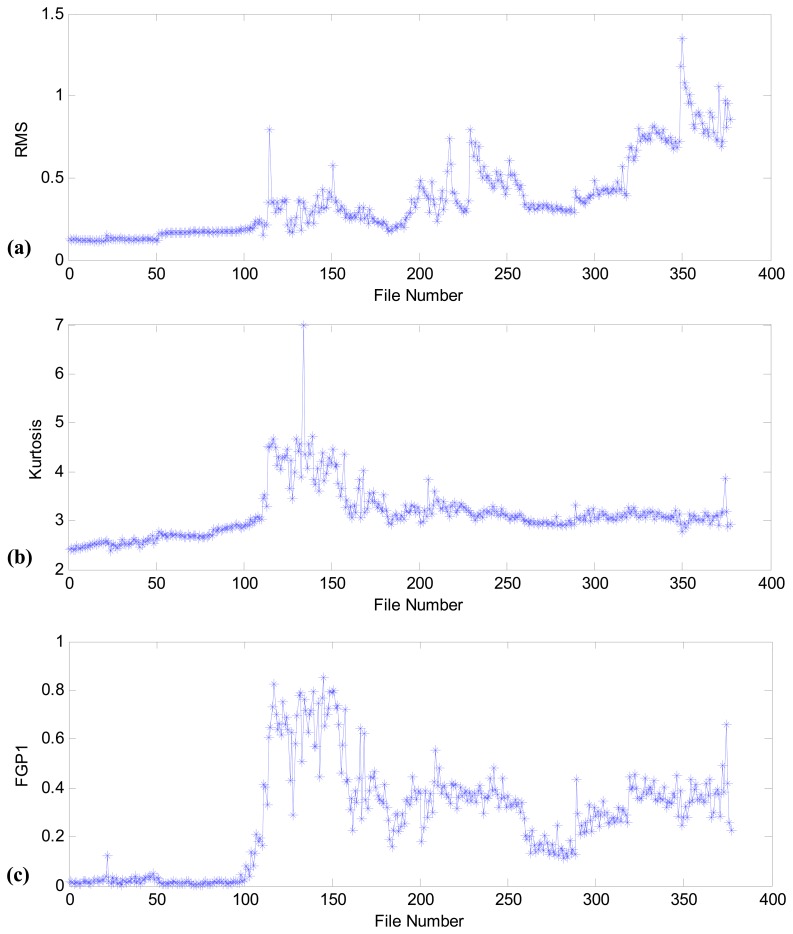
Comparison results of RMS, kurtosis, and FGP1 in fan bearing health assessment: (**a**) RMS; (**b**) kurtosis; (**c**) FGP1.

Figure 9 shows the health assessment results of RMS, kurtosis, and FGP1 using fan bearing vibration data. It is obvious that the performance of HCI (in Figure 8) is much better than these health indicators. For example, based on the results presented in Figure 9, incipient fan bearing failures may occur at file number 110. However, according to the spectrum analysis results presented in Figure 8, the time of incipient failure should be around file number 98. Furthermore, the trend in fan bearing degradation cannot be observed in Figure 9, and the health assessment methods using RMS, kurtosis, and FGP1 cannot be used for the further research on fan bearing prognosis.

## 5. Conclusions

Cooling fans are commonly used in microelectronics. In order to ensure high reliability in air-cooled electronic systems, it is necessary to conduct research on the life expectancy and health assessment of cooling fans. Fan bearing failure is a major failure mode that causes excessive vibration, noise, reduction in rotation speed, locked rotor, and failure to start, among other problems, which may result in an electronic system's malfunction and lower the electronics reliability.

This paper presents a coherent solution for the health assessment of cooling fan bearings. The method utilizes the comblet concept. A health indicator was proposed based on the techniques of comblet filtering and exponentially weighted moving average. An accelerated life test was conducted on a cooling fan to simulate fan bearing degradation. The recorded vibration data were used to validate the proposed method. To demonstrate the performance of the proposed method, a comparative study was conducted between the proposed HCI and the commonly used methods of RMS, kurtosis, and FGP1. Based on the analysis results, the HCI can detect incipient fan bearing failures, and the bearing degradation process can be captured by the proposed method.

The work presented in this paper provides a promising method for cooling fan bearing health evaluation and prognosis. With this method, the critical failure of a cooling system can be avoided, and the reliability of electronic systems can be guaranteed. Furthermore, the proposed solution may also be used in generic bearing health evaluation and prognosis, which is currently the focus of prognostics and health management of mechanical systems.

## Figures and Tables

**Table 1.** The geometric specifications of the fan bearing in this experiment.

Number of rolling elements, *n*	6
Mean diameter of rolling element, *d*	1.59 mm
Pitch diameter of bearing, *D*	5.5 mm
Contact angle, *γ*	10.4°

**Table 2.** The bearing characteristic frequencies.

Ball spin frequency (BSF)	127.178 Hz
Ball pass frequency, inner race (BPFI)	308.242 Hz
Ball pass frequency, outer race (BPFO)	171.758 Hz
Fundamental train frequency (FTF)	28.626 Hz

**Table 3.** The parameters of each comblet filter with *f_s_*=25.6 kHz.

**Filter type**	**Central Frequency,***f*_0_	**Rotation Variation Parameter,***c*	**Half Central Bandwidth,** bw	**Number of Comb Teeth,***n*
Comblet filter: BSF	127.178 Hz	2%	1.272 Hz	100
Comblet filter: BPFI	308.242 Hz	2%	3.082 Hz	41
Comblet filter: BPFO	171.758 Hz	2%	1.718 Hz	74
Comblet filter: FTF	28.626 Hz	2%	0.286 Hz	447
